# Adenylylation of Tyr77 stabilizes Rab1b GTPase in an active state: A molecular dynamics simulation analysis

**DOI:** 10.1038/srep19896

**Published:** 2016-01-28

**Authors:** Manuel P. Luitz, Rainer Bomblies, Evelyn Ramcke, Aymelt Itzen, Martin Zacharias

**Affiliations:** 1 Physics Department T38, Technische Universität München, 85748 Garching, Germany; 2 Center for Integrated Protein Science Munich, Technische Universität München, Department Chemistry, 85748 Garching, Germany

## Abstract

The pathogenic pathway of *Legionella pneumophila* exploits the intercellular vesicle transport system via the posttranslational attachment of adenosine monophosphate (AMP) to the Tyr77 sidechain of human Ras like GTPase Rab1b. The modification, termed adenylylation, is performed by the bacterial enzyme DrrA/SidM, however the effect on conformational properties of the molecular switch mechanism of Rab1b remained unresolved. In this study we find that the adenylylation of Tyr77 stabilizes the active Rab1b state by locking the switch in the active signaling conformation independent of bound GTP or GDP and that electrostatic interactions due to the additional negative charge in the switch region make significant contributions. The stacking interaction between adenine and Phe45 however, seems to have only minor influence on this stabilisation. The results may also have implications for the mechanistic understanding of conformational switching in other signaling proteins.

The orchestration of intracellular protein interaction networks requires tight temporal and spatial regulation in order to maintain homeostasis and to react to changing environmental conditions. Small Ras-like GTPases (guanosine triphosphate phosphohydrolases), also referred to as G-proteins, play a pivotal role in the coordination of intracellular signaling by acting as binary molecular switches^1^. The discrimination between the active and inactive state is achieved by differential co-factor binding: GTPases are in the active (“on”) state when bound to guanosine triphosphate (GTP), but inactive (“off”) when complexed with guanosine diphosphate (GDP). Signaling is promoted by the binding and recruitment of effector proteins that specifically interact with the active state of the GTPase. Due to their fundamental role in coordinating signaling it is not very surprising that many intracellularly replicating bacterial pathogens have evolved with mechanisms to interfere with GTPase activities and thereby promote their survival^2^. Among several activity modulating strategies, the covalent attachment of additional functional groups (also referred to as posttranslational modifications (PTMs)) appears to be particularly prominent and interesting. We and others have previously observed that the pathogenic bacterium *Legionella pneumophila* covalently modifies the human G-protein Rab1b via the posttranslational attachment of adenosine monophosphate (AMP) from adenosine triphosphate (ATP) to the side chain of residue Tyr77 with the help of the bacterial protein DrrA (also known as SidM). This enzymatic adenylylation (also termed AMPylation) exploits the intracellular vesicle transport system of its host cell^3^. It has been observed that adenylylation of small GTPases can abrogate the interaction with GAPs^3^ or downstream effectors^4^,^5^. In particular, adenylylation of Rab1 blocks the access of the human GAP TBC1D20 and the Legionella GAP LepB *in vitro*^3^,^6^,^7^. Thus, Rab1 adenylylation appears to stabilize the G-protein in the active GTP-state by inhibiting GAP-mediated GTP-hydrolysis. It is, however, less obvious whether the adenylylated Rab1 protein also maintains an active conformation that is in principle capable of interacting with GTP-state specific cellular factors. The activity state of a GTPase is communicated to interaction partners mainly via two highly important regulatory regions that are referred to as switch I and switch II. In the inactive GDP-bound state, these regions are structurally disordered but they become highly conformationally restrained in the active GTP-bound form. Interacting molecules very sensitively probe the switch conformations and thus can bind specifically either the GDP- or the GTP-state. Interestingly, the adenylylated residue Tyr77 of Rab1 is located in the switch II region and consequently the question arises as to how this modification may affect the configurational ensemble of the switch II and/or switch I regions. The X-ray structure of the AMP-Rab1:GTP complex revealed a stacking interaction of the adenine base of the adenylylated Tyr77 of switch II with a highly conserved phenylalanine (Phe45) side chain^3^. This interaction may suggest that Tyr77-adenylylation fixes switch II in a defined and active–state like conformation. Furthermore, we have observed recently that the deadenylylation reaction of AMP-Rab1 by SidD is independent of the nucleotide-state of the G-protein, i.e. AMP-Rab1:GDP and AMP-Rab1:GTP show no difference in their substrate properties^8^.

This observation is a very astonishing finding since most GTPase interacting molecules very sensitively discriminate between the GDP- and GTP-states by binding only to the inactive or the active switch conformations, respectively. The lack of discrimination of SidD between the GDP- and GTP-states of AMP-Rab1 may therefore suggest that the switch regions are locked in identical conformations. Consequently, adenylylation of Rab1b could force the switch regions into the active conformation even if the protein is actually in the GDP-state possibly due to the stacking interaction with the Phe45 residue observed in the crystal structure.

In order to elucidate this mechanism we performed extensive continuous Molecular Dynamics (MD) and Umbrella Sampling (US) based free energy simulations to compare the influence of adenylylation on Rab1b conformational states bound to either GTP or GDP. The simulations indicate a stabilizing effect of the Tyr77 adenylylation on the active form of Rab1b even in the presence of GDP. In addition, electrostatic energy analysis of conformational ensembles close to the active states vs. states representing the inactive form reveals that electrostatic interactions make the major favorable contribution to the active state stabilization in the presence of the Tyr77 adenylylation. The simulation study indicates that stabilizing effects of side chain modifications in GTPases (not necessarily close to the GTP/GDP binding site) might not only be mediated by contacts but also indirectly e.g. by electrostatic interactions. The result may also have important implications for understanding the influence of other modifications on signaling proteins.

## Results

### Molecular Dynamics simulations and *in vitro* deadenylylation assay on Rab1b

In order to elucidate the influence of the bound nucleotide and adenylylation of Tyr77 on the conformational flexibility and stability of Rab1b we first performed a series of continuous (c)MD-simulations in explicit solvent. The simulations were started from the crystal structure of the GTP bound form with native or adenylylated residue Tyr77 (i.e. AMP covalently attached to the OZ atom of Tyr77). Starting structures in complex with GDP with or without Tyr77 modification were generated in silico by removing the corresponding atoms from the crystal structure. In all simulations the protein structure remained overall close to the starting conformation with an overall backbone root-mean-square deviation (RMSD) of <0.2 nm with respect to the crystal structure (Figure S1 , supp. material). The calculated root-mean-square-fluctuation (RMSF) of the switch I and II regions showed larger fluctuations in the GDP vs GTP complexes but no sign of unfolding, e.g. towards the inactive form in the presence of GDP (Figure S2, supp. material).

**Figure 1:**
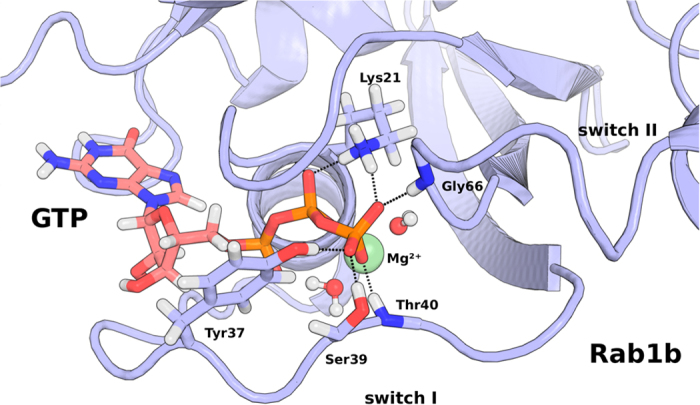
Noncovalent interaction network of GTP bound to Rab1b observed during Molecular Dynamics simulations. GTP is depicted as atom-color-coded sticks while Rab1b is indicated as blue cartoon. The bound magnesium ion is shown as a green sphere. Rab1b residues forming bonds with GTP are shown as blue sticks. The *γ*-phosphate group of GTP forms 3 hydrogen bonds with switch I residues Tyr37, Ser39, and Thr40 of Rab1b. One additional bond is formed between switch II residue Gly66 and GTP. The nucleotide is further stabilized in the binding pocket by two salt bridges formed with core residue Lys21. Two water molecules build a charge shielding shell around Mg^2+^ ion and are tightly bound during all the simulations.

The Mg^2+^ ion stayed close to the initial placement in simulations with either GTP or GDP bound to Rab1b. In simulations with GTP two water molecules persisted in the close vicinity of Mg^2+^ exposing the oxygen atom which carries the negative partial charge to the Mg ion (Fig. 1). This induced a local shielding effect of the two positive charges by the dipole field of the water molecules. These water molecules were also found in the crystal structure of Rab1b^9^. The cavity arising from the hydrolysis of GTP to GDP (i.e removal of the *γ*-phosphate from GTP) was filled with a third water molecule which persisted throughout all simulations with GDP bound to Rab1b. Residues Ser39, Thr40 and Tyr37 located in the switch I region formed persistent H-bonds with the *γ*-phosphate during the simulation in line with the X-ray structure^9^. Also in agreement with experiment, one persistent H-bond between *γ*-phosphate and the backbone of switch II residue Gly66 was observed.

**Figure 2:**
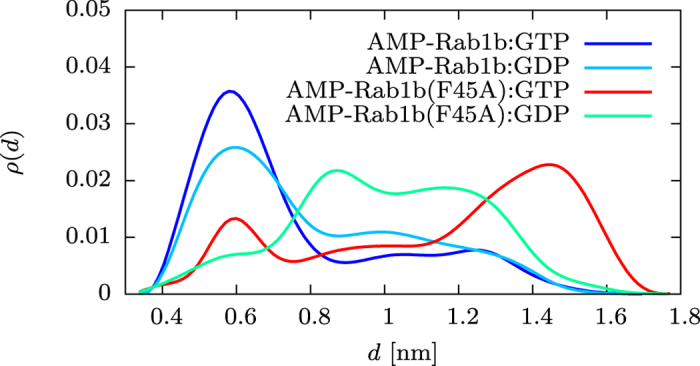
Probability distribution of sampled distances between the C*β*-atom of residues 45 and the adenine double ring structure of AMP-Tyr77 during cMD simulations. A short distance (below 0.7 nm) indicates a stacking interaction between the adenine and residue 45. Sampling of larger distances corresponds to non-contacting states.

**Figure 3:**
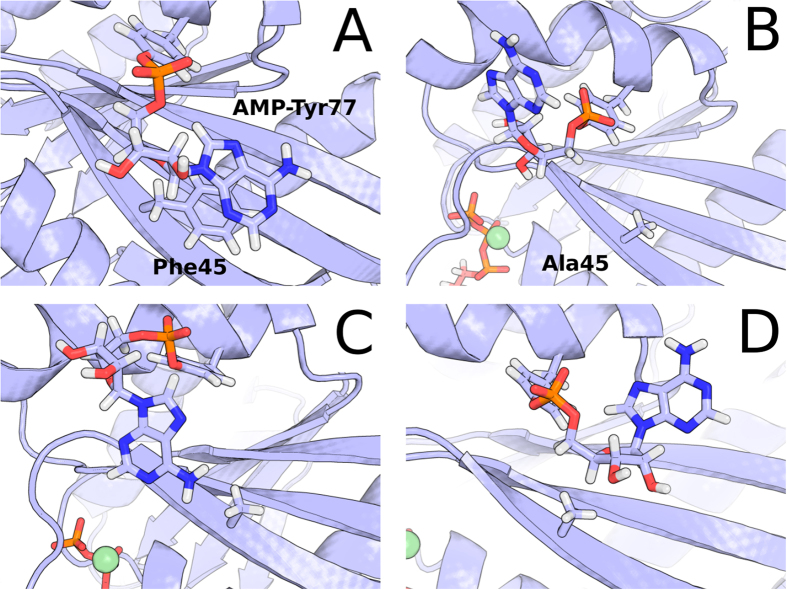
Stacking interactions between the adenine double ring of adenylylated Tyr77 with the phenyl ring of Phe45 observed during 600 ns continuous MD simulation of AMP-Rab1b:GTP. Clustering of AMP-Tyr77 orientations indicated stacked conformations (largest conformational cluster) occurring in 74% of the whole simulation trajectory (**A**). The stacking interaction, however, is greatly reduced in the simulation of the F45A mutation. The decoupled AMP sidechain showed high flexibility and visited various states at increased distance to Ala45 during a 600 ns simulation of the mutant. The three largest clusters are depicted (**B**–**D**).

The X-ray structure of the AMP-Rab1b:GppNHp complex revealed a planar packing of the highly conserved Phe45 phenyl with the adenine double ring of adenylylated Tyr77 which suggests a functionally relevant *π*-stacking interaction^3^. Based on this suggestion additional 600 ns simulations of the adenylylated Rab1b mutant F45A (Rab1b(F45A)) in the presence of either GDP or GTP were performed and compared with simulation results of wild type AMP-Rab1b. Again, neither the GTP nor the GDP bound case resulted in significant structural changes in the switch regions during the simulation time (see Figure S1 , supp. Material). However, the conformational sampling of the adenine base attached to Tyr77 is altered by the F45A mutation: A histogram plot of distances between the C*β*-atom of residue 45 and the adenine double ring of AMP-Tyr77 reveals a significant loss of the stacking interaction between the Ala45 side chain and the adenine ring in the Rab1b(F45A) mutant compared to wild-type (both in the GTP or GDP bound case, Fig. 2). Cluster analysis indicated a dominating cluster for AMP-Rab1b with about 74% of all frames representing a stacking interaction between Phe45 and AMP when GTP was bound and slightly less with bound GDP (see Figs 2 and 3A). For the F45A mutant the clustering revealed that the AMP modification gains conformational freedom visiting a broader range of configurations (Fig. 3B–D: three of the five largest clusters). Importantly, the average distance between AMP group and residue 45 is considerably larger in case of the F45A mutation compared to the wild type (Fig. 2) incompatible with an effective stacking interaction.

**Figure 4:**
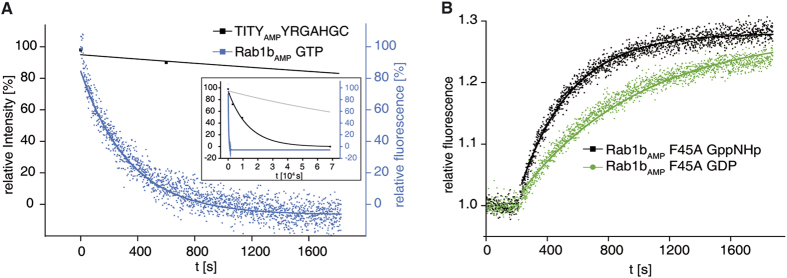
Kinetics of deadenylylation (**A**) Deadenylylation of AMP-Rab1b:GTP and the adenylylated peptide TITY_AMP_YRGAHGC by SidD revealing a tremendous preference for the adenylylate protein. Demodification of AMP-Rab1b:GTP (5 μM) or peptide (50 μM) were initiated with catalytic amounts of SidD (50 nM or 500 nM, respectively). Deadenylylation was monitored using the change in intrinsic tryptophan fluorescence (AMP-Rab1b:GTP) or by quantifying reaction products on reversed phase chromatography (peptides). Data were fitted to a single exponential function. Inset: Comparison of reaction progress on long time scales. For comparison, the reaction progress curve for 5 μM peptide-AMP with 50 nM SidD (grey) was simulated as described in methods. (**B**) Deadenylylation of Rab1b AMP-Rab1b(F45A):GDP (green) and AMP-Rab1b(F45A):GppNHp (black) by SidD. Deadenylylation of 1 μM Rab1b was initiated by addition of 100 nM SidD and monitored via intrinsic tryptophan fluorescence.

In order to probe its relevance for the switch mechanism we performed enzymatic deadenylylation reactions of preparatively modified AMP-Rab1b. We have observed previously that the deadenylylation enzyme SidD does not discriminate between GDP- and GTP-bound AMP-Rab1b^8^. However, SidD shows much weaker activity toward the synthetic adenylylated switch II peptide TITYAMPYRGAHGC in comparison to AMP-Rab1b:GTP 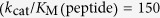 M^−1^s^−1^ vs. 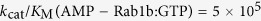 M^−1^s^−1^) (Fig. 4)^10^. This observation is a strong indication that SidD requires the recognition of specific structural elements in Rab1b rather than merely binding to the adenylylated tyrosine. The structural specificity of SidD in particular and since GTPase-binding proteins and enzymes usually discriminate very sensitively activity states by probing the conformations of the switch regions, the hypothesis was derived that adenylylation locks both GDP- and GTP-forms in the same conformation. If the Phe45-adenine interaction was relevant for this locking effect, a F45A substitution would be expected to promote conformational segregation and thus affect SidD catalytic rates (resulting in preference of AMP-Rab1(F45A):GTP over AMP-Rab1(F45A):GDP). However, the differences in catalytic deadenylylation of AMP-Rab1(F45A):GppNHp vs AMP-Rab1(F45A):GDP were negligible (Fig. 4) and thus similar (active) conformations of the switch regions in the AMP-Rab1(F45A) proteins are expected. Consequently, the Phe45 may not contribute significantly to rigidifying the conformations of adenylylated Rab1 in both activity states, suggesting that other molecular effects are predominant in locking the conformational states.

### Free energy calculation of switch region unfolding reveals stabilization by adenylylation

**Figure 5:**
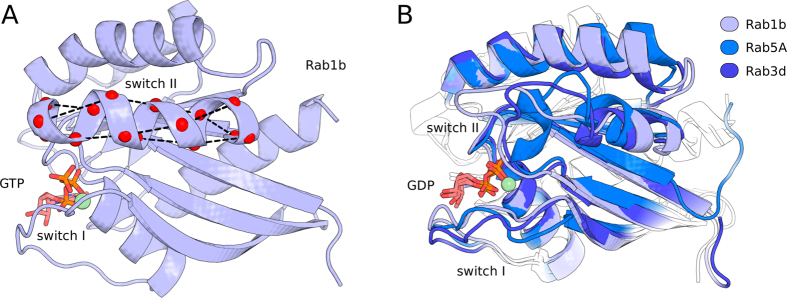
(**A**) Illustration of the set of distances which contributed to the dRMSD bond network in order to bias the unfolding of the switch II region. Contributing atoms are highlighted as red spheres, the distances are shown as dashed lines. Rab1b is shown in its active conformation taken from X-ray structure (PDB code 3NKV) but without adenylylation. (**B**) Superimposed structures of Rab1b homologs human RAS-related proteins Rab3d (PDB code 2GF9) and Rab5a (PDB code 3CLV) in GDP bound form with a representative inactive state snapshot from dRMSD US simulations. The snapshot agrees qualitatively with unfolded features of switch I and switch II regions observed in the Rab1b GDP bound homologs.

**Figure 6:**
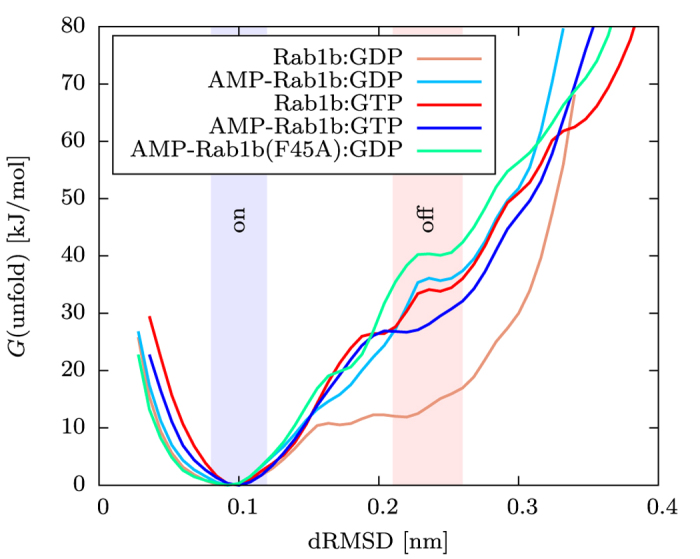
The effect of different modifications in Rab1b on the unfolding free energy of switch II along the dRMSD coordinate. Only the inactive Rab1b:GDP form has a significantly lower unfolding free energy of the switch II region compared to the other Rab1b modifications. The removal of the Phe45 stacking interaction with adenylylated Tyr77 by F45A mutation shows no notable difference in the unfolding free energy profile compared to the AMP-Rab1b:GDP version.

In order to directly probe the effect of chemical modification and/or mutation of Rab1b on the transition between active and inactive states (conformational ensembles), we performed Umbrella Sampling (US) free energy simulations. This approach permits to induce conformational transitions associated with the active and inactive states using a penalty potential to unfold the switch region during simulations. It also allowed us to calculate the associated change in free energy (also termed potential of mean force: PMF) for the transition and how it depends on adenylylation and on bound nucleotide. As a reaction coordinate for the US simulations the mean deviation of a set of distances within the switch II region from the active state was employed (dRMSD coordinate, illustrated in Fig. 5 and explained in detail in the Methods section). A small reference dRMSD results in sampling of conformations close to the active GTPase conformation whereas unfolding of the switch region is induced with increasing reference dRMSD (modified in 14 umbrella windows from 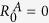 nm to 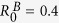 nm). In order to improve the convergence of the US simulations frequent replica exchanges between neighboring US windows were allowed (H-REUS technique, see Methods). The calculated PMF showed reasonable convergence after 80 ns of data gathering time in each US window (Figure S3, supp. material). The active state ensemble is represented by conformations close to the minimum of the calculated free energy curve (Fig. 6) at small dRMSD (below 0.15 nm). Already at dRMSD >0.15 nm the switch region starts to unfold. The definition of a dRMSD range for the inactive state was based on the comparison with GDP bound GTPase X-ray structures and their associated dRMSD values (0.2 nm for the set of distances, which is also close to the plateau regime observed in the calculated free energy curves, Fig. 6). Note, that during the US simulations along the dRMSD coordinate an ensemble of conformations at the regime of the inactive state was sampled (Figure S4). Hence, the simulations support the view that the inactive state is not represented by a single stable conformation but compatible with various unfolded conformations. In all systems the unfolding process resulted in an increase in free energy along the reaction coordinate (Fig. 6). However, in case of an unmodified Rab1b:GDP the free energy increase was significantly (about 14 kJ/mol) smaller than in case of a bound GTP. The calculated free energy changes were also tested with respect to changes in the force constants used to control the dRMSD deviation from a reference during the US simulations (supp. Information, Figure S5). For increasing or lowering the force constant by a factor of 2 or 4, respectively, almost the same free energy change (within ~3 kJ mol^−1^, supp. Information, Figure S5) was obtained indicating the calculated PMFs are robust with respect to changes in the force constants used in the US simulations. In addition, the presence of the adenylylated Tyr77 appears to stabilize the active state of the GTPase for both the GTP and GDP bound cases (Fig. 6). The calculated free energy change for the AMP-Rab1b:GDP case is similar to the Rab1b:GTP curve. For Rab1b:GDP a consecutive unfolding was found starting with the switch II helix unfolding in lower dRMSD replica and followed by switch I at higher dRMSD indicating a coupling of both conformational regimes. Unfolding simulations of switch II revealed that the major conformational rearrangement occurred in the N-terminal part of the helix between residues Gly66 and Tyr77 (see MD-snapshot in Fig. 5). In addition to the wild type Rab1b protein, free energy simulations were also performed on the F45A mutation of AMP-Rab1b:GDP. Surprisingly, the unfolding characteristics of the F45A mutant were similar to the AMP-Rab1b:GDP simulation indicating that the observed transient stacking between AMP and Phe45 (observed in the cMD simulations) may not be of dominant importance for stabilizing the active state (Fig. 6). The results of the free energy simulations are summarized in Table 1.

### Electrostatic effects of adenylylation affect Rab1b conformations

Since the proposed stacking interaction between Phe45 and the adenine base appeared not to be of major significance for stabilizing the switch region, we aimed at analyzing the electrostatic effects resulting from the presence of AMP on Rab1b conformations. The adenylylated Tyr77 residue is located in the switch II region relatively far from the GTP binding site. In order to investigate the influence of long-range electrostatic interactions we compared the electrostatic energy of Rab1b in the active and inactive states. The electrostatic energies were calculated for an ensemble of conformations extracted from the AMP-Rab1b:GDP umbrella sampling simulations with dRMSD values of around 0.1 nm representing the active state. A conformational ensemble of unfolded switch II region with dRMSD around 0.25 nm represented the inactive conformational ensemble. Snapshots were taken from the trajectories every 0.6 ns skipping the initial 12 ns to account for equilibration. Electrostatic contributions can be split into Coulomb interactions between atoms belonging to Rab1b in the different ensembles (termed Coulomb contributions) and secondary interactions of the protein atoms with the surrounding solvent (termed electrostatic solvation or reaction field contribution). Both average contributions can differ in the active versus inactive ensembles depending on distances between charges (Coulomb contribution) and accessibility to solvent (solvation or reaction field contribution). The total electrostatic energy is given as a sum of these two contributions.

The Coulomb contributions and solvent induced reaction field contributions were calculated with the finite-difference Poisson-Boltzmann (FDPB) approach and a continuum solvent representation (see Methods for details). In order to focus on the influence of adenylylation and to minimize the numerical error of the FDBP solutions, the calculations were repeated after replacing the adenylylated residue 77 with an unmodified Tyr residue in the active and inactive ensembles. This procedure does not account for possible differences in the sampled ensembles between adenylylated and unmodified Rab1b but allows to focus directly on the adenylylation effect.

While the direct Coulomb interactions favor the unfolded structure of AMP-Tyr77 compared to unmodified Tyr77 by about −2.6 kJ mol^−1^, the inverse effect was observed for the reaction field contribution with about 22.4 kJ mol^−1^ (Table 2). In total, the wildtype Rab1b:GDP inactive ensemble of switch II is favored electrostatically by 19.9 kJ mol^−1^ compared to AMP-Rab1b:GTP. The calculated difference in electrostatic energy is consistent with the PMF unfolding curves (suggesting a free energy difference of about 20 kJ mol^−1^, Table 1) and provides a possible energetic explanation for the stabilizing effect of AMP on the switch regions. Although not at the focus of the present study we also compared the mean electrostatic energy of Rab1b:GTP versus Rab1b:GDP in active versus inactive ensembles using the same procedure. In this case the calculations predicted a strong favorisation of the inactive ensemble vs. active ensemble of the Rab1b:GDP complex relative to the Rab1b:GTP complex by 29.0 kJ mol^−1^ (Table 2). This result predicts that electrostatic interactions stabilize an active state ensemble (relative to the inactive state ensemble) considerably more in the presence of GTP versus GDP (in agreement with the experimental observation). Note, that the same trend was also found for calculations using an internal dielectric constant of 2 or 4 for the protein (see supp. Material Table S1) which in part accounts for the possible relaxation of the protein charge distribution upon removal of the Tyr77 modification or switch from GTP to a bound GDP in the trajectory analysis. It is also consistent with the calculated trend from the free energy simulations which predicted a relative stabilization free energy of 18.8 kJ mol^−1^ (Table 1). Note, that an agreement is not necessarily expected because other energetic and entropic contributions also contribute to the relative stabilities of inactive and active conformational ensembles. These contributions are accounted for the in free energy simulations but not in the electrostatic energy calculations.

## Discussion

The molecular mechanism how adenylylation of residue Tyr77 within the switch II region affects the active and inactive conformations of Rab1b has not been addressed previously. Here, we employed MD and free energy simulations to characterize the conformational flexibility and stability of Rab1b in different GDP/GTP states in dependence of Tyr77 adenylylation. On the time scale of 600 ns of our continuous MD simulations similar flexibility patterns of the different Rab1b nucleotide complexes were found. The observed hydrogen bonding pattern and location of water molecules was compatible with available experimental structures^3^. In case of Tyr77 adenylylation, the adenine group of AMP stacked on the neighboring Phe45 for a significant fraction of the simulation time but also other states with fully solvent exposed AMP group were sampled. A F45A substitution in silico resulted in the elimination of this *π*-stacking interaction and increased the conformational flexibility of Tyr77-AMP. The modified and unmodified Rab1b:GDP complexes showed generally larger fluctuations in the switch I and switch II regions compared to GTP-bound complexes. However, no spontaneous unfolding of the switch regions characteristic for an inactive conformation in the presence of GDP was observed on the time scale of the cMD simulations. This is not surprising since the estimated time scale of such changes is in the range of tenth of seconds^11^,^12^,^13^.

In order to still calculate the free energy change associated with a transition to the inactive state we employed US based on a dRMSD coordinate that allows gradual unfolding of the switch region and transition to conformations representing the inactive state. The sampled inactive conformations agreed qualitatively well with switch II helix and switch I loop regions found in related Ras-GTPases (X-ray structures of the GDP bound form of Rab proteins , PDB codes 2GF9, 3CLV and 4Q21, Fig. 5)^14^. The simulations predicted in all cases an increase of the free energy towards unfolding of the switch region. Although this may support a view that Rab1b behaves as a non-classical GTPase there is are many experimental studies that demonstrate that Rab1b indeed operates by a classical GTPase-mode. This is supported by the crystal structure of a homologous Rab1a GTPase (more than 95% sequence identity to Rab1b) in the presence of GDP (PDB code 2FOL) which indicates an unfolded and disordered structure of the switch regions. In addition, it has been shown that several Rab1 effector proteins (such as Mical-1, Mical-2C, Mical-3C, Golgin84, GM130, p115, Rabaptin5) favor the GTP-bound state over the GDP state^15^,^16^,^17^,^18^,^19^,^20^,^21^,^22^. In addition, the GDP-specific Rab-binding protein GDI has been shown in several instances that it does not bind to the GTP-state but only interacts with the inactive GDP Rab1b form^23^,^24^. These results indicate that Rab1b significantly differs in its conformations between the GDP- and GTP-states since effectors (GTP-specific) and GDI (GDP-specific) discriminate unambiguously between the activity states. Our US simulations along the dRMSD coordinate did not reveal a single compact conformation representing the inactive state but suggest a largely disordered more solvent exposed ensemble of conformations. It is well known that current molecular mechanics force fields overestimate the stability of compact states even of disordered proteins^25^,^26^. In turn this might cause an artificial over-stabilization of the folded compact states of the switch regions. Nevertheless, the calculated free energy change associated with a transition from an active to an inactive state was significantly smaller in the presence of GDP compared to GTP confirming that the relative change for the transition is in qualitative agreement with the experimental observation.

Furthermore, the US simulations showed that the adenylylation of Tyr77 stabilizes the active state relative to the inactive state regardless of GDP or GTP binding. The free energy costs of unfolding the switch region (AMP-Rab1:GDP to Rab1:GDP) were similar to the level obtained for Rab1b:GTP (Rab1:GTP to Rab1:GDP). This result agrees with an interpretation that Tyr77 adenylylation could lock Rab1b in an active conformation in the AMP-modified form^3^,^8^. Hypothetically, the stacking interaction of the AMP group with the Phe45 observed in the experimental X-ray structure^3^ and during the cMD simulations may be involved in this stabilization. However, a similar calculated free energy change associated with the unfolding process was obtained for a F45A mutation in silico, suggesting that base stacking is not the major contribution to conformational stabilization of AMP-Tyr77. An analysis of the electrostatic energy of the active and inactive states revealed that the presence of the adenylylation destabilizes the ensemble of the inactive conformations mainly due to a reduced electrostatic solvation (solvent reaction field) contribution. In combination with our observation, that the geometric coordination of the adenine group of AMP does not impair the stabilization of the active state ensemble we assume, that mainly the inclusion of an additional negative charge in switch II located at the phosphate group of AMP modifies the electrostatic field such that the reaction of surrounding dielectric solvent molecules inhibits a transition to the inactive ensemble.

The combination of MD simulations and *in vitro* enzymatic activity assays suggest that adenylylation of Rab1 at switch II tyrosine 77 significantly changes conformational transitions of the GTPase domain. As a consequence, the G-protein surprisingly adopts an active-like conformation in both nucleotide states and thus AMP-modification may in this instance uncouple Rab-activation from the conventional cyclic GDP-GTP-binding. Generally, the effects of PTMs on the conformations of small GTPases using structural or computational methods are only poorly characterized. In one study, Kalbitzer and coworkers used 1H and 31P nuclear magnetic resonance (NMR) spectroscopy to investigate the consequences of Thr35-glycosylation of H-Ras to gain insight into structural consequences of this particular PTM^27^. Their work supported an earlier model from crystallographic studies of glycosylated H-Ras that suggested a disordering of switch I due to interference with Mg^2+^ coordination and Tyr32-nucleotide interactions^28^. In this respect, glycosylation impaired effector protein binding by promoting a disordered switch I state and by sterically interfering with protein-protein interactions. Rab1 adenylylation appears to have the opposite effect and actually promotes the active conformations of the switch region. It will be interesting to see whether other modifications of small GTPases (i.e. adenylylation of Cdc42 on Thr35 or Tyr32, phosphocholination of Rab1b on Ser76^4^,^5^,^29^) will have similar conformational consequences.

Our study indicates that chemical modifications located at a distance from the nucleotide binding site of a Rab1b protein may considerably affect the conformational equilibrium of active and inactive states. In addition to contacts in the neighborhood of the modification, long range electrostatic interactions contribute significantly to stabilizing an active signaling state which may also be relevant for understanding the influence of other modifications on the signaling mechanism in other signaling proteins.

## Methods

### Molecular Dynamics Simulation Setup

The crystal structure of the Rab1b protein with an Adenosinmonophosphate (AMP) moiety covalently attached to OZ atom of Tyr77 in complex with the GTP analog Phosphoaminophosphonic Acid Guanylate Ester (GNP) (PDB code 3NKV) served as start structure for the simulations. The nitrogen atom in GNP bridging the beta to the gamma phosphate group was replaced by an oxygen atom which resulted in the AMP-Rab1b:GTP complex model structure. For simulations on unmodified Rab1b or bound to GDP the AMP modification and/or the *γ*-phosphate group were removed to create start structures of the Rab1b:GTP, AMP-Rab1b:GDP, and Rab1b:GDP complexes, respectively. All simulations were performed with the GROMACS molecular dynamics software suite release version v4.6^30^,^31^. The Amber ff99sb-ILDN forcefield^32^ was used for the protein and parameters for guanosine nucleotides GDP and GTP were taken from Carlson *et al.*^33^. Partial charges for the adenylylated Tyr77 residue were calculated using Gaussian03^34^ with B3LYP^35^,^36^ with the 6–31G* basis set level^37^ and a total charge of −1*e* following the restraint electrostatic potential (RESP) protocol^38^. The antechamber program^39^ of the AmberTools13 package^40^ in combination with the general amber forcefield GAFF^41^ was used to assign atom types and bonded parameters. The system topologies were finally prepared with the tleap tool from the AmberTools13 software package and then translated to Gromacs topology files via acype^42^. To render the systems charge neutral sodium ions were added and the protein was solvated with the TIP3P water model^43^ in a truncated octahedron box with periodic boundary conditions and a minimum distance of 1 from solute to the box boundaries.

### Simulation protocol

After the setup procedure, energy minimization with the steepest decent algorithm was performed until one convergence criterion of either 20 k steps or a maximum force below 100 kJ mol^−1^ nm^−1^ was reached. The minimization was followed by two short equilibration runs of each 150 ps duration at a time step of 1 fs, primarily in the NVT and then in the NPT ensemble. All heavy backbone atoms were restraint in space with a harmonic potential at force constant of 1000 kJ mol^−1^nm^−2^ to avoid conformational rearrangements whilst equilibration. The equations of motions were solved according to the leap-frog integrator (MD) and the Particle Mesh Ewald (PME) algorithm was used to calculate long range electrostatics^44^ with a grid interpolation up to the order of 6 (4 in NPT and production run) and FFT grid spacing of 0.12 nm. The Lennard–Jones interactions were switched to zero after 1.0 nm with a cutoff value of 1.1 nm for both Lennard–Jones and real space electrostatic interactions. The temperature was adapted to a reference of 298 k with the velocity rescale^45^ algorithm and pressure in the NPT equilibration phase was controlled with the Berendsen barostat^46^ to equal 1.01 bar. For production runs the time step size was increased to 2 fs and the Parinello–Rahman barostat was applied^47^. A long range dispersion correction for energy and pressure was applied to account for the error introduced by truncated Lennard-Jones interactions. The LINear Constraint Solver^48^ with a coupling matrix extension order of 12 (4 in production run) constrained the bond lengths involving H atoms.

### Stacking interaction between Phe45 and AMP-Tyr77 sidechains

Sidechain conformations of AMP-Tyr77 were clustered for the 600 ns trajectories of systems Rab1b:GTP, Rab1b:GDP, Rab1b(F45A):GTP, and Rab1b(F45A):GDP in order to characterise the prevalence of the *π*-stacking interaction between adenine double ring of AMP-Tyr77 with the phenyl ring of Phe45. The protein trajectories were aligned before clustering of the AMP-Tyr77 sidechain was performed using the single linkage method with a RMSD cutoff of 0.07 nm as implemented in g_cluster of the GROMACS toolchain. Additionally the distance between C*β* atom of residue 45 and the center of mass of the adenine double ring has been monitored over time and a histogram was calculated by splitting the observed distance range in 100 bins (Fig. 2).

### Free energy simulations based on the root mean square deviation of a set of intramolecular distances

The root mean square deviation of a set of distances (dRMSD) obtained during the simulation with respect to the same set of distances in a reference structure was used as reaction coordinate. In contrast to Cartesian RMSD this reaction coordinate does not require a superposition step and is invariant under rotation. An additional advantage is that it is possible to include only subsets of distances that are spatially separated in the dRMSD coordinate (e.g. only local short range distances) which would require several superposition operations if using the Cartesian RMSD as reaction coordinate. The collective variable is defined as the RMS sum over a set of *N* interatomic distances 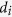 with a reference distance 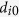 (eq. 1).


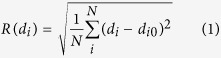


The spring like biasing potential which emerges from the dRMSD (eq. 2) is expanded to two reference dRMSD values 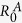 and 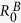 which are linearly connected over a coordinate λ and allows for the calculation of a Potential of mean Force (PMF) curve along this coupling coordinate.


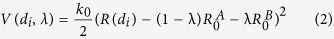


The biasing potential was used to perform umbrella sampling (US) free energy simulations along the reaction coordinate λ. The simulations were carried out with an in-house implementation of the dRMSD potential in the GROMACS v4.6 software. In order to improve the sampling of relevant conformational states along the dRMSD coordinate during US simulations replica exchanges between simulations of neighboring λ values were introduced (REUS-technique). Replica exchanges were attempted every 1000 steps alternating between odd and even indexed neighboring replica pairs to avoid that one system state could swap with more than one replica window per exchange step. The force constant for the harmonic dRMSD potential function was set to a value of 1000 kJ mol^−1^ nm^−2^ and the reference dRMSD was changed in equidistant steps from 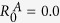 nm to 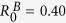 nm in the replica windows. The number of 14 replica windows was adjusted in test simulations such that exchange rates higher than 30% were recorded. In order to seed the reference distances for the distance pairs which accounted to the dRMSD, average distances were calculated from the 600 ns cMD simulations. The PMF along the dRMSD coordinate was calculated with the WHAM^49^ algorithm implemented by Grossfield *et al.*^50^, using 100 bins and a tolerance of 10^−5^ kJ.

### Electrostatic energy calculations

The electrostatic energy of Rab1b conformations in different modification and in the presence of different nucleotides was calculated using the finite-difference Poisson Boltzmann (FDPB) approach implemented in the APBS software package^51^. Structures representing the active state (represented by replica window with average dRMSD ≈ 0.1 nm with respect to the crystal start structure) and the inactive state (replica with dRMSD ≈ 0.23 nm) were obtained as snapshots from the trajectories every 600 ps skipping the initial 12 ns to account for equilibration. All water molecules and surrounding ions were removed and the linearized FDPB was solved for each snapshot with the APBS software package^51^ using a two-stage focusing (initial boundary conditions were calculated with the multiple Debye-Hückel option) and a final grid spacing of 0.3 Å. The divalent Mg^2 + ^was included in the continuum electrostatics calculation as it has a significant effect on the local electrostatic field of the nucleotide binding site and the nearby switch regions and was found to be conserved in its binding site throughout all simulations. A dielectric constant of 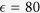 for solvent was used^52^ for protein in water was used. A permittivity of 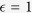 in the protein was used to directly compare with the explicit solvent simulations which employ a vacuum permittivity and implicitly include orientational polarisation effects since a trajectory of conformations was analysed. Electrostatic energy calculations for the AMP-Rab1b:GDP ensemble in inactive and active states were repeated after replacing the adenylylated residue 77 with tyrosine (resulting in Rab1b:GDP structures) in order to estimate the electrostatic contribution of the AMP modification to the switch opening. This procedure minimizes all errors of electrostatic energy calculations which arise from the grid representation of the molecules because the investigated structures differ only in the presence or absence of the AMP group but not in the coordinates of all other atoms (placement relative to the grid). The same protocol was applied to inactive and active conformations of the Rab1b:GTP ensembles and subsequent in silico mutation to Rab1b:GDP by deletion of the *γ*-phosphate in GTP, in order to give an estimate for the electrostatic contribution of GTP vs. GDP to the switch I/II stability.

### Protein Expression and Purification

Rab1b proteins, SidD_37−350_ and DrrA_8−533_ were produced as described previously^3^,^8^,^23^. In brief, SidD_3−350_ and DrrA_8−533_ were cloned in a pET19 vector with N-terminal Hexahistidine-tag (His_6_-tag) and a tobacco etch virus cleavage site. Protein production in *E. coli* BL21 CodonPlus (DE3)-RIL cells was induced by addition of 0.5 mM IPTG overnight at 20 °C. Purification was achieved by Nickel affinity chromatography including cleavage of the His_6_-tag and final gel filtration in 20 mM HEPES pH 7.5; 100 mM NaCl; 2 mM DTE; 1 mM MgCl_2_. Rab1b_3−174_ proteins were produced in a pMAL vector with N-terminal His_6_-MBP tag and a tobacco etch virus cleavage site. Rab1b mutant proteins were generated by site-directed mutagenesis. Protein production in *E. coli* BL21 CodonPlus (DE3) cells was induced by addition of 0.5 mM IPTG overnight at 20 °C. Purification was achieved by Nickel affinity chromatography including cleavage of the His_6_-MBP-tag and final gel filtration in 20 mM HEPES pH 8.0; 50 mM NaCl; 2 mM DTE; 1 mM MgCl_2_; 10 μM GDP.

### Preparative nucleotide exchange

Nucleotide exchange of Rab1b proteins was performed as described earlier^3^. In brief, Rab proteins were incubated with 5 mM ethylendiamintetraacetid acid (EDTA) and a 20 times molar excess of nucleotide at 25 °C for at least 2 hours in exchange buffer (20 mM HEPES pH 8.0; 50 mM NaCl, 2 mM DTE). Excess nucleotide was removed by using a PD-10 column (GE Healthcare) in storage buffer (20 mM HEPES pH 8.0; 50 mM NaCl, 2 mM DTE; 1 mM MgCl_2_; 10 μM nucleotide). Completeness of the exchange was verified by reversed phase HPLC analysis.

### Preparative Adenylylation

Preparative adenylylation of Rab1b was performed as described previously^3^. In brief, Rab1b was incubated with a 2.5 molar excess of ATP and an 0.01 molar ratio of DrrA_8−533_ at room temperature. Completeness of the reaction was verified by mass spectrometry. The modified protein was purified by size exclusion chromatography (20 mM HEPES pH 8.0; 50 mM NaCl, 2 mM DTE; 1 mM MgCl_2_; 10 μM GDP).

### Deadenylylation of Peptide-AMP by SidD

Deadenylylation of the switch II peptide TITY_AMP_YRGAHGC by SidD_37–350_ was analyzed in a time-dependent manner by reversed phase chromatography using an Aeris C4 widepore column (Phenomenex) on a Shimadzu HPLC system. Peptide (50 μM) was incubated with 0.5 μM SidD at 25 °C and subjected to reversed phase HPLC analysis at indicated time points. A binary gradient of 100% H_2_O containing 0.01% trifluoracetic acid (TFA) and 100% acetonitrile containing 0.01% TFA from 5 to 25% acetonitrile at 1 ml min^−1^ flow rate was used to elute peptides. Peptides were detected by measuring tyrosine absorption at 274 nm. The progress curve of 5 μM peptide-AMP by 50 nM SidD was calculated in OriginPro v8.6G (OriginLab, Northampton, MA) using a 10 times lower rate constant.

### Deadenylylation assay

Deadenylylation by SidD_37−350_ was measured using the change in tryptophan fluorescence as reported previously^53^ in a Fluoromax-3 spectrophotometer (HORIBA Jobin Yvon) (excitation at 297 nm; emission at 340 nm). Start of the deadenylylation reaction was induced by adding 50 nM and 100 nM of SidD_37−350_ to 5 μM Rab1b_3−174_ Q67A and 1 μM Rab1b_3−174_ F45A in 20 mM HEPES pH 7.5, respectively; 5 mM NaCl; 5 mM MgCl_2_; 2 mM DTE at 25 °C.

## Additional Information

**How to cite this article**: Luitz, M. P. *et al.* Adenylylation of Tyr77 stabilizes Rab1b GTPase in an active state: A molecular dynamics simulation analysis. *Sci. Rep.* **6**, 19896; doi: 10.1038/srep19896 (2016).

## Supplementary Material

Supplementary Information

## Figures and Tables

**Table 1:** Calculated relative free energy differences of unfolding the switch II region with respect to Rab1b:GDP (ΔΔ*G*) and absolute unfolding free energy (Δ*G*) in kJ/mol from US simulations.

System^a^	ΔΔ*G*^b^	Δ*G*^c^
AMP-Rab1b:GDP	20.1	32.1
AMP-Rab1b(F45A):GDP	25.6	37.6
AMP-Rab1b:GTP	14.7	26.7
Rab1b:GTP	18.8	30.8
Rab1b:GDP	—	12.0

The absolute unfolding free energies Δ*G* were calculated by integrating the PMF for the active regime (corresponding dRMSD range 0.08–0.12 nm) and the inactive regime (dRMSD range 0.21–0.26 nm) and substracting the values. The free energy of unfolding for the Rab1b:GDP case was again substracted from Δ*G* values to get relative ΔΔ*G*.

^a^values in [kJ/mol].

^b^unfolding free energy relative to Rab1b:GDP.

^c^unfolding free energy.

**Table 2:** Electrostatic contributions to the mean energy difference of inactive vs. active conformational ensembles of Rab1b:GDP in the presence or absence of the adenylylation at Tyr77 based on FDPB calculations (see Methods for details).

Difference electrostatic unfolding energy^a^	ΔΔ*Ε*_coulomb_	ΔΔ*Ε*_reaction–field_	ΔΔ*Ε*_total_
AMP-Rab1b:GDP–Rab1b-GDP	−2.6 ± 1.8	22.4 ± 10.4	19.8 ± 12.2
Rab1b:GTP–Rab1b-GDP	−1.5 ± 1.4	30.5 ± 10.4	29.0 ± 11.8

A positive ΔΔ*Ε* indicates a stronger favorisation of the active form (relative to inactive) for the adenylylated variant (equivalent to a relative stabilization of the inactive ensemble vs active ensemble in the absence of adenylylation). The energetic contributions are split into two contributions for direct Coulomb interactions and the electrostatic solvation (reaction field) term, respectively. The top row shows the relative electrostatic stabilization of the wildtype vs. the adenylylated Rab1b (both with GDP bound). The second row indicates the corresponding electrostatic energy differences for active vs inactive conformations in case of GTP vs. GDP bound to Rab1b (indicating an electrostatic stabilization of the active conformational ensemble by the presence of GTP compared to GDP).

^a^values are given in [kJ/mol].
