# Supplementary material for: Adenylylation of Tyr77 stabilizes Rab1b GTPase in an active state: A molecular dynamics simulation analysis
Source: Sci Rep. 2016 Jan 28;6:19896. doi: 10.1038/srep19896 (PMC4730224; doi:10.1038/srep19896)
Supplement: Supplementary Information [file srep19896-s1.pdf]

Supplemental Materials: Adenylylation of Tyrosine 77 stabilizes Rab1b GTPase in an active state: A molecular dynamics simulation analysis

Manuel P. Luitz,[1] Rainer Bomblies,[1] Evelyn Ramcke,[2] Aymelt Itzen,[2] and Martin Zacharias[1,*]

[1] *Physics Department T38, Technische Universität München, 85748 Garching, Germany*
[2] *Center for Integrated Protein Science Munich, Technische Universität München, Department Chemistry, 85748 Garching, Germany*

Convergence of dRMSD simulations

In order to estimate the required simulation time to obtain well converged potential-of-mean-force (PMF) curves for simulations with a dRMSD biasing potential on the switch II region, we tested different simulation times of up to 120 ns per dRMSD interval (window) with 14 replica windows on the unmodified Rab1b:GDP system biasing the unfolding of switch II along the dRMSD coordinate. The cumulative PMF along the dRMSD coordinate was plotted every 10 ns (figure S3). We found that the PMF change was smaller than 1 kJ mol^{-1} per 10 ns or 0.1 kJ mol^{-1} ns^{-1} after approximately 80 ns. Accordingly, following these benchmark results we chose a simulation time of 80 ns for all dRMSD H-REUS simulations. The convergence criterion of 0.1 kJ mol^{-1} ns^{-1} for the change in PMF per simulation time was met by all dRMSD PMF simulations within the 80 ns .

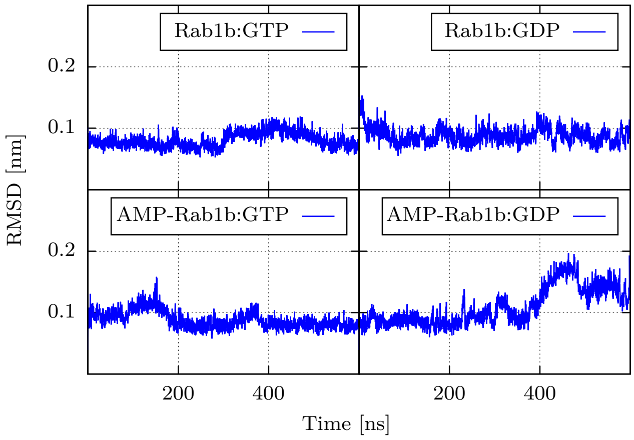

Figure S1. Root-mean-square deviation (RMSD) of protein backbone with respect to the AMP-Rab1b:GppNHp X-ray structures of four cMD simulations for different Rab1b systems. The flexible five N- and C-terminal residues were excluded from the RMSD calculations, respectively. The initial conformations were well conserved throughout the 600 ns simulations which is reflected in a RMSD below 0.2 nm for all simulations.

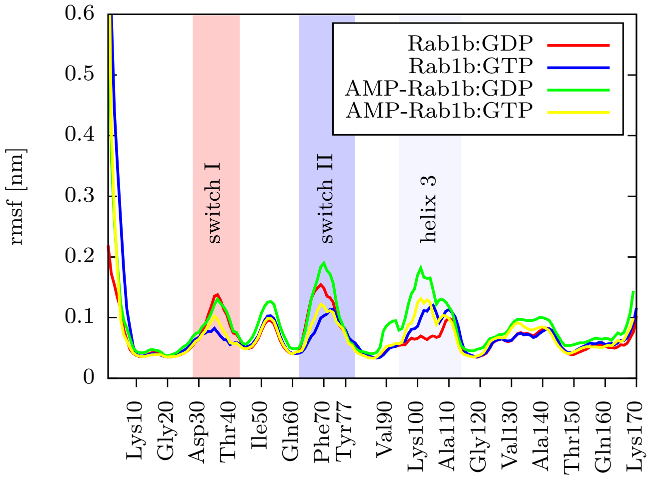

Figure S2. Average root mean square fluctuations (RMSF) of Rab1b C-α atoms in GTP and GDP bound form taken from 600 ns MD simulations. The difference of both curves highlights the change in flexibility depending on the present nucleotide. The removal of the γ-phosphate destabilizes switch I and II regions due to the loss of hydrogen bonds network and the shift in the electrostatic field.

Difference electrostatic unfolding energy [a]	$\epsilon_{\text{protein}}$	$\Delta\Delta E_{\text{coulomb}}$	$\Delta\Delta E_{\text{reaction-field}}$	$\Delta\Delta E_{\text{total}}$
AMP-Rab1b:GDP − Rab1b-GDP	1	-2.6 ± 1.8	22.4 ± 10.4	19.8 ± 12.2
AMP-Rab1b:GDP − Rab1b-GDP	2	-1.3 ± 0.9	16.2 ± 9.4	14.9 ± 10.3
AMP-Rab1b:GDP − Rab1b-GDP	4	-0.7 ± 0.5	9.9 ± 7.6	9.2 ± 8.1
Rab1b:GTP − Rab1b-GDP	2	-0.8 ± 0.7	18.9 ± 13.5	18.1 ± 13.2
Rab1b:GTP − Rab1b-GDP	1	-1.5 ± 1.4	30.5 ± 10.4	29.0 ± 11.8
Rab1b:GTP − Rab1b-GDP	4	-0.4 ± 0.4	11.6 ± 7.7	11.2 ± 8.1

[a] $\Delta\Delta E$ values are given in [kJ/mol]

Table S1. Electrostatic contributions to the mean energy difference of inactive vs. active conformational ensembles of Rab1b:GDP in the presence or absence of the adenylylation at Tyr77 based on FDPB calculations (see Methods for details). Energies have been calculated for three different dielectric constants in protein ($\epsilon = 1, 2, 4$). The reaction field stabilizes the active conformation more when AMP or GTP are present and the effect is reproducible for different protein permittivities. The reduction in calculated energy differences with increasing ϵ results the increasing similarity of solvent and solute permittivity. The conformations that were used for FDPB calculations were extracted from simulations in explicit solvent using a dielectric permittivity of $\epsilon = 1$ which therefore is the correct permittivity for comparison with MD results.

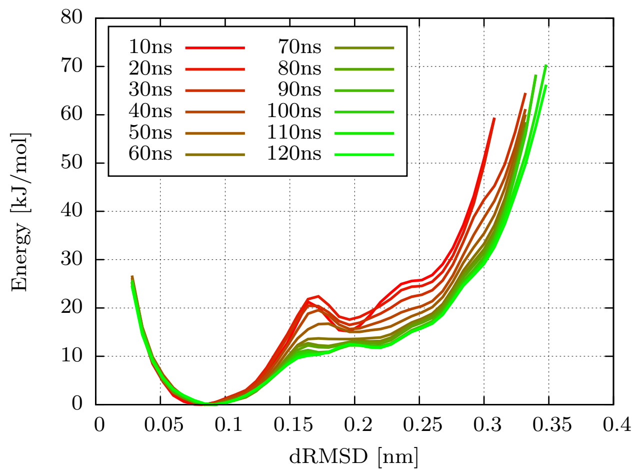

Figure S3. Convergence of PMF along the global dRMSD coordinate for AMP-Rab1b:GDP system over time. The cumulative PMF is plotted every 10 ns and the initial 5 ns are excluded from the calculation for equilibration. PMF does not change by more than 0.1 kJ mol^{-1} ns^{-1} after approximately 80 ns.

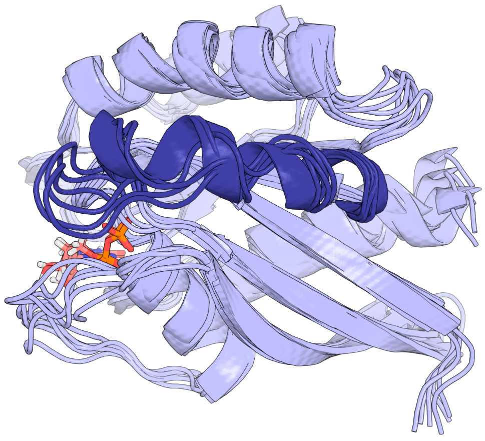

Figure S4. An ensemble of conformations representing the transtition from active to inactive state. Switch II is colored in dark blue, the rest of Rab1b is colored lightblue. Snapshots are taken from different replicas of the dRMSD H-REUS simulations.

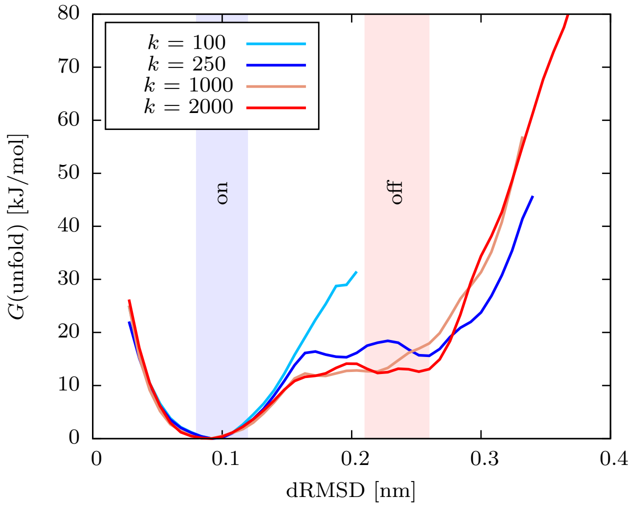

Figure S5. Different force constants for the dRMSD biasing potential (equation 2) have been tested for unfolding of switch II region exemplarily on the Rab1b:GDP system. Force constants k are given in [kJ mol^{-1} nm^{-2}]. The biasing potential was increased in 14 steps from a reference value of $R_0^A = 0.0$ to $R_0^B = 0.65$ (in production runs $R_0^B = 0.40$ was used). Note that the effective force constant acting on a dRMSD atom pair is $k_{\text{eff}} = k/N$ with the number of atom pairs $N = 11$ resulting from the derivative of the biasing potential after d_i. The resulting PMFs converge for forces constants over 250 kJ mol^{-1} nm^{-2}. At even lower force constant of 100 kJ mol^{-1} nm^{-2} the biasing potential is unable to unfold switch II region resulting in no sampling of the inactive configurations.
